# First person – Gigi Lau and Nick Barts

**DOI:** 10.1242/bio.044321

**Published:** 2019-05-15

## Abstract

First Person is a series of interviews with the first authors of a selection of papers published in Biology Open, helping early-career researchers promote themselves alongside their papers. Gigi Lau and Nick Barts are co-first authors on ‘ Detection of changes in mitochondrial hydrogen sulfide *in vivo* in the fish model *Poecilia mexicana* (Poeciliidae)’, published in BiO. Gigi is a post-doctoral fellow in the lab of Dr Sjannie Lefevre at the University of Oslo, Norway, adaptive variation in mitochondrial function. Nick is a PhD candidate in the lab of Dr Michael Tobler at University of British Columbia, Canada, investigating the physiological mechanisms of adaptation to extreme environmental conditions in fishes.

**What is your scientific background and the general focus of your lab?**


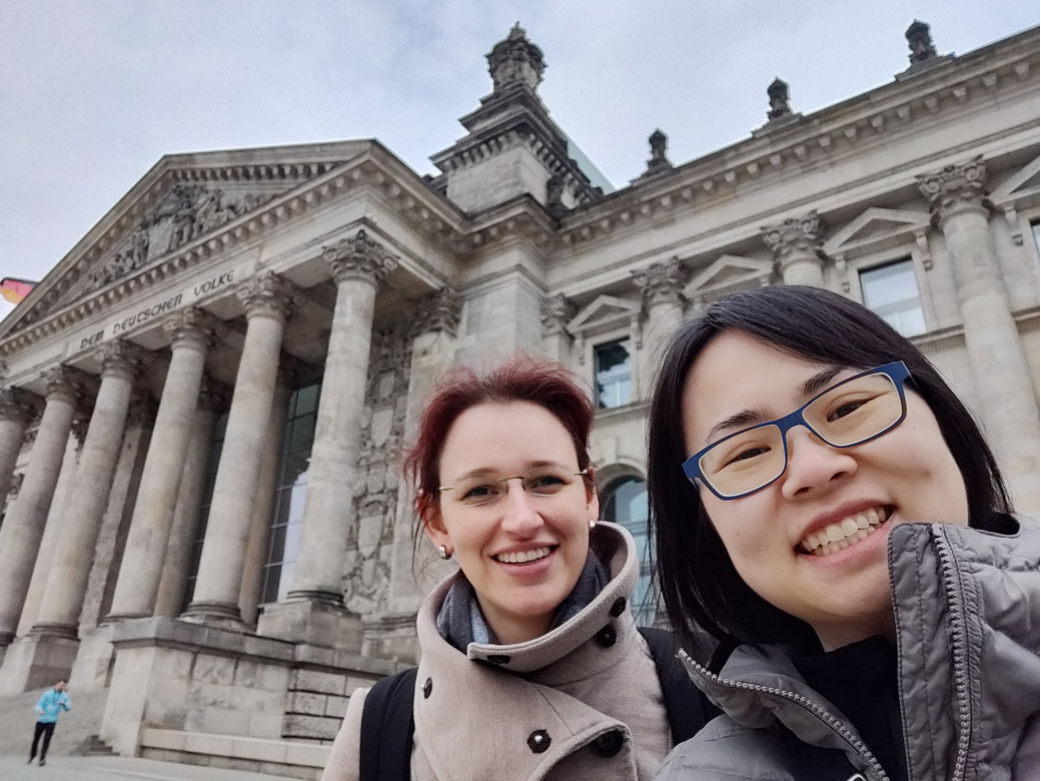


**Gigi Lau (right) and** **Sabine Arndt (left)**

GL: I am a comparative physiologist. I completed my graduate degrees at the University of British Columbia and am now a postdoctoral research fellow at the University of Oslo. I am broadly interested in how animals are able to deal with environmental challenges, specifically focusing on oxygen limitation. I find this really interesting because as humans, we are unable to survive without oxygen, but some animals are able to do so for extended periods of time. I have been lucky to work on a range of interesting animal models that live in diverse oxygen habitats, such as marine intertidal fishes, naked mole rats that live in borrows, and turtles and crucian carp that endure anoxia overwintering. What is exciting to me is to find commonalities in how they respond to hypoxia/anoxia. My primary focus is on adaptive mechanisms at the level of mitochondria where oxygen is used for aerobic metabolism.


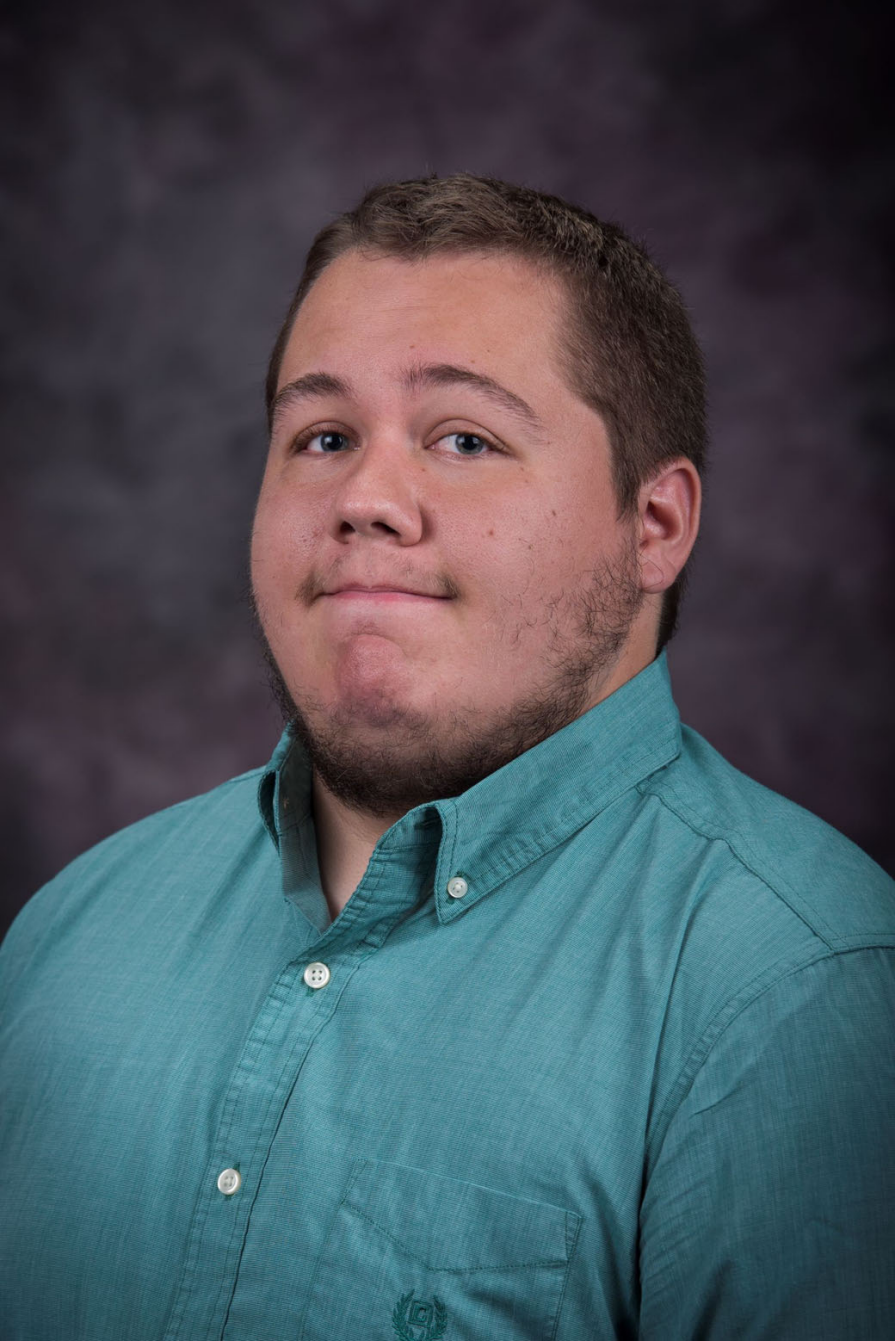


**Nick Barts**

NB: I often find myself asking how and why animals do the things they do. This curiosity led me down my path to become a comparative physiologist. I'm fascinated by the ability of animals to tolerate extreme environmental conditions and challenges, and I am currently completing my doctoral degree at Kansas State University investigating the mechanisms that allow some species of fish to tolerate hydrogen sulfide-rich habitats. My research looks at adaptive mechanisms across biological levels of organization, but I am particularly interested in the role mitochondria plays in adaptation.

**How would you explain the main findings of your paper to non-scientific family and friends?**

Hydrogen sulfide (H_2_S) is a naturally occurring compound that is toxic at moderately high levels. Its toxicity is due to its ability to directly inhibit aerobic metabolism, which takes play within cells at subcellular organelles called mitochondria. Inhibition of aerobic metabolism would threaten an animal's ability to generate the cellular energy required to survive. Despite this, some organisms are capable of living in H_2_S-rich environments, but the strategies they use to tolerate these conditions are not well understood. Evidence suggests that mitochondria, which not only are the primary target of H_2_S toxicity, play an important role in regulating H_2_S levels within cells. Therefore, understanding the effects of H_2_S on mitochondria will help us understand how organisms may have adapted to living in these ‘toxic’ habitats. There are, however, very few reliable tools for measuring mitochondrial H_2_S in animals. Arndt and colleagues developed a specialized probe called MitoA that can be used to measure H_2_S levels within live animals, and was used in mice studies prior. Here, we have adapted this technique for use in fish, specifically cavefish from a population that is tolerant to high environmental H_2_S. We hope this method can be used for studying organisms that vary in H_2_S tolerance in order to shed light on adaptive strategies for dealing with high environmental H_2_S.

“Understanding the effects of H_2_S on mitochondria will help us understand how organisms may have adapted to living in these ‘toxic’ habitats.”

**What are the potential implications of these results for your field of research?**

Most vertebrates cannot tolerant high levels of H_2_S, however, H_2_S has been found to play important roles in normal cellular function, and disruption of H_2_S balance has been linked to a number of human pathologies. With MitoA, we not only have a tool to measure changes in mitochondrial H_2_S levels in isolated mitochondria or cell preparations to study H_2_S metabolism, but it can also be used in whole-animal studies where the study organism can be exposed to a variety of different conditions.

**What has surprised you the most while conducting your research?**

The ability of organisms to survive, even thrive, in what we humans consider to be extreme environmental conditions never ceases to amaze. The diverse adaptive strategies animals use to deal with the same environmental stressor is fascinating.

NB: One of the most surprising things for me is the ability of biomedical and evolutionary biology researchers to work together to answer complicated questions in both fields, such as using techniques developed for measuring health of cells to quantify potential differences in naturally occurring populations' ability to tolerate stress.

GL: How tolerant these fish are of high H_2_S was very surprising! They really did not seem bothered at all. Also, how quickly this collaboration came together, to me, was surprising. I was on a research visit to the MRC Mitochondrial Biology Unit for a different collaborative project with Mike (Murphy), and happened to be talking to Sabine (Arndt) about her project regarding MitoA. I had just been working on a population of the H_2_S-tolerant cavefish at UBC in collaboration with Michi (Tobler) and mentioned this to Sabine. It did not take much convincing to get everyone across the different lab groups on-board!

“The ability of organisms to survive, even thrive, in what we humans consider to be extreme environmental conditions never ceases to amaze.”

**What changes do you think could improve the professional lives of early-career scientists?**

NB: Working as an early-career scientist can be daunting. There is increasingly more, and there still needs to be more, recognition that there are more career outcomes and alternatives to academia than just research and publications. Teaching and scientific communication are both important factors in increasing scientific literacy and engagement of the general public. Early on in graduate school, expectations between mentors and graduate students should be communicated clearly such that students can obtain the skills necessary for their desired STEM career and set realistic goals for the duration of the degree.

GL: Having a good peer group and incredibly supportive mentors has been very important to me as an early career researcher, especially since I moved to a new country for my postdoctoral position. Also, the thing I most enjoy about being a scientist is to be able to collaborate and think broadly, so I think more research funding opportunities to do this as an early-career scientist is important in order to establish independency.

**Figure:**
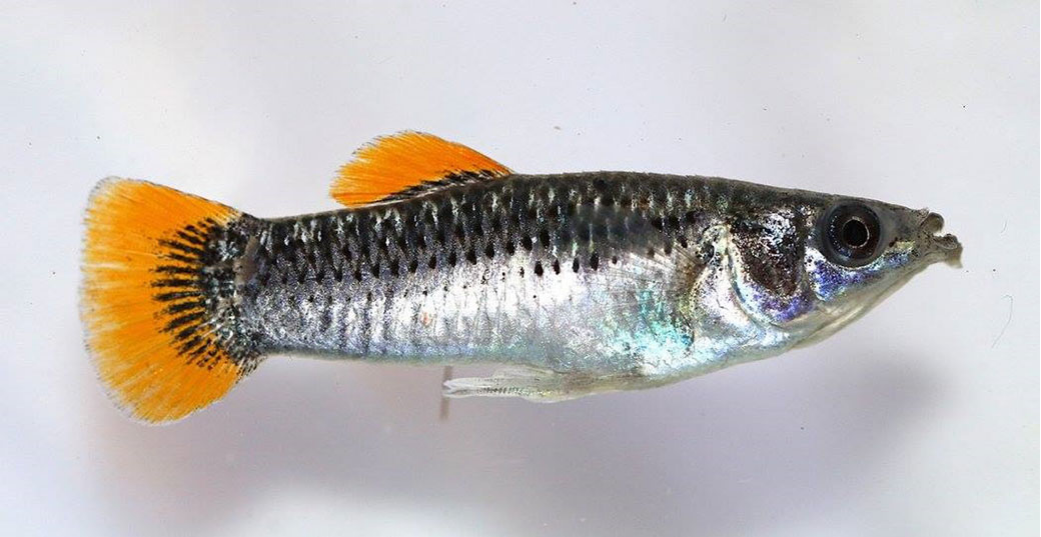
***Poecilia mexicana*, the Atlantic** **molly, adapted to hydrogen sulfide-rich springs in southern Mexico. These fish are an ideal study organism for investigating adaptations to extreme environments.**

**What's next for you?**

NB: My passion is science. Although I am unsure of exactly what I will be doing upon completing my PhD, I know that research and science education will be a big part of it.

GL: For now, I will continue to enjoy my time as a postdoctoral fellow at the University of Oslo studying anoxia-tolerant crucian carp and aim to stay in academia. It would be great to find an academic job, if not, I would still be happy tinkering in a government or industry lab somewhere.
